# Fibroblast Growth Factor 9 is expressed by activated hepatic stellate cells and promotes progression of hepatocellular carcinoma

**DOI:** 10.1038/s41598-020-61510-4

**Published:** 2020-03-11

**Authors:** Tatjana Seitz, Kim Freese, Peter Dietrich, Wolfgang Erwin Thasler, Anja Bosserhoff, Claus Hellerbrand

**Affiliations:** 1 Institute of Biochemistry, Emil-Fischer-Zentrum, Friedrich-Alexander-University Erlangen-Nürnberg, Erlangen, Germany; 2 Medical Clinic 1, Department of Medicine, University Hospital Erlangen, Friedrich-Alexander-University, Erlangen, Germany; 3 Hepacult GmbH, Planegg/Martinsried, Germany; 4 Comprehensive Cancer Center (CCC) Erlangen-EMN, Erlangen, Germany

**Keywords:** Hepatocellular carcinoma, Hepatic stellate cells

## Abstract

Hepatocellular carcinoma (HCC) is closely associated with liver fibrosis. Hepatic stellate cells (HSC) and cancer-associated myofibroblasts are key players in liver fibrogenesis and hepatocarcinogenesis. Overexpression of fibroblast growth factor (FGF) receptors contributes to HCC development and progression. This study aimed to elucidate the role of FGFs in the HSC-HCC crosstalk. Analysis of the expression of the fifteen paracrine FGF-members revealed that FGF9 was only expressed by HSC but not by HCC cells. Also in human HCC tissues, HSC/stromal myofibroblasts were identified as cellular source of FGF9. High expression levels of FGF9 significantly correlated with poor patient survival. Stimulation with recombinant FGF9 induced ERK- and JNK-activation combined with significantly enhanced proliferation, clonogenicity, and migration of HCC cells. Moreover, FGF9 significantly reduced the sensitivity of HCC cells against sorafenib. Protumorigenic effects of FGF9 on HCC cells were almost completely abrogated by the FGFR1/2/3 inhibitor BGJ398, while the selective FGFR4 inhibitor BLU9931 had no significant effect. In conclusion, these data indicate that stroma-derived FGF9 promotes tumorigenicity and sorafenib resistance of HCC cells and FGF9 overexpression correlates with poor prognosis in HCC patients. Herewith, FGF9 appears as potential prognostic marker and novel therapeutic target in HCC.

## Introduction

Hepatocellular carcinoma (HCC) is one of the most aggressive malignancies and one of the most common causes of cancer death worldwide^1^. In most cases, HCC is diagnosed in already advanced stages, and currently, there are very limited options for HCC treatment. The multi-tyrosin kinase inhibitor sorafenib is the first-line treatment for advanced-stage HCC patients, however, the benefits are at best modest and transient^1,2^. Therefore, better understanding of the drivers of HCC development and progression and new therapeutic targets are highly needed.

HCC is strongly associated with liver fibrosis and cirrhosis, suggesting that the environment in which HCC arises may influence cancerogenesis^3^. The activation of hepatic stellate cells (HSC) into extracellular matrix (ECM)-producing myofibroblasts is the key event of hepatic fibrosis^4^. Several studies using genetic cell fate mapping have provided strong evidence that HSC are also the major precursors of myofibroblasts in the HCC microenvironment^3^, and that HSC/stromal myofibroblasts promote HCC development and progression^3,5^. Therefore, a better understanding of the role of HSC and their crosstalk with HCC cells may provide new therapeutic options for the treatment of HCC.

Fibroblast growth factor (FGF) signaling plays an important role in the regulation of many biological processes such as development, cell proliferation and differentiation, and its dysregulation is reported in different types of diseases including cancers^6^. The FGF family comprises 22 proteins that can be classified into intracrine, endocrine and paracrine factors. Paracrine FGFs can be further subclustered into five subfamilies (FGF1/2; FGF3/7/10/22; FGF4/5/6; FGF8/17/18 and FGF9/16/20). All FGFs interact with heparan sulfate proteoglycans. However, paracrine and endocrine FGFs show different affinity to these matrix components, which results in a predominantly local action of paracrine FGFs near the place of secretion^6^. The FGFs signal through four transmembrane tyrosine kinase FGF receptors (FGFR) namely FGFR1, FGFR2, FGFR3 and FGFR4^6^.

Several alterations in FGF-signaling have been found to affect liver carcinogenesis^7^. Aberrant expression of the endocrine FGF19 and its high affinity FGFR4 contributes to HCC progression^8^. Furthermore, overexpression of FGFR2 and FGFR3 contributes to HCC development and metastasis^9,10^, further suggesting that FGF-signaling plays an important role in HCC.

While most previous studies had focused on the role of FGFRs in HCC, the aim of the present study was to get a deeper understanding of the expression and tumorigenic effects of different FGFR-ligands with a focus on paracrine FGFs and their role in the HSC-HCC crosstalk.

## Results and Discussion

### FGF9 expression in HCC

**Figure 1:**
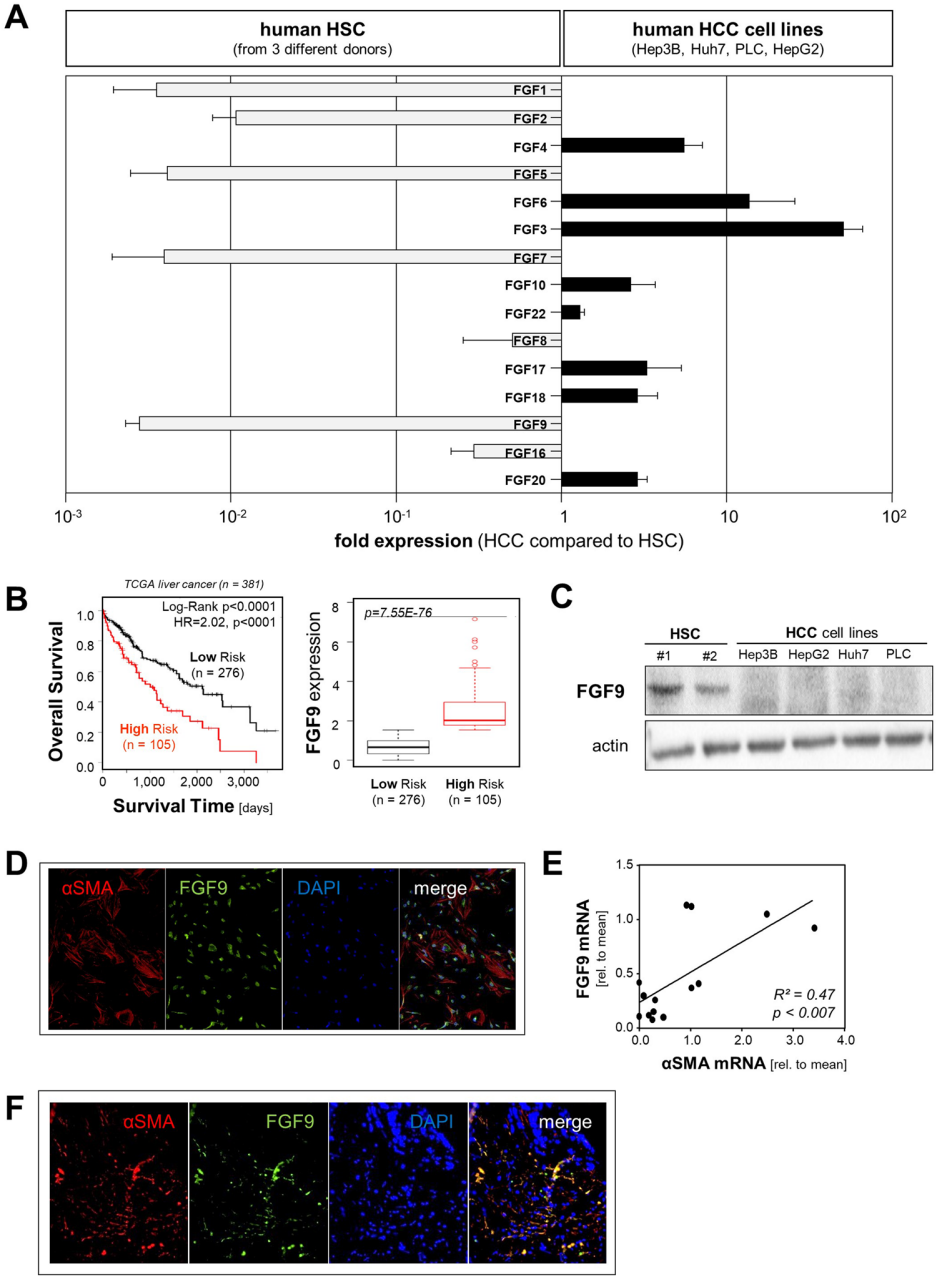
Analysis of paracrine FGFs expression with focus on FGF9 in HCC. (**A**) Fold mRNA expression levels of paracrine FGFs (subfamilies 1 (FGF1 and FGF2), 4 (FGF4, FGF5 and FGF6), 7 (FGF3, FGF7, FGF10 and FGF22), 8 (FGF8, FGF17, FGF18) and 9 (FGF9, FGF16, FGF20)) in four human HCC cell lines (Hep3B, Huh7, PLC, HepG2) compared to expression in primary human hepatic stellate cells (HSC) from three different donors. (**B**) **“**SurvExpress-Biomarker validation for cancer gene expression” database analysis of FGF9 expression (right panel) and corresponding Kaplan-Meier curve for overall survival (left panel) in “TCGA liver cancer” dataset. Computational stratification of patients into “Low Risk” and “High Risk” groups was based on prognostic index and according to the “Maximized Risk Groups” algorithm. **(C)** Western blot analysis of FGF9 protein levels in primary human hepatic stellate cells (phHSC) and human HCC cell lines (Hep3B, HepG2, Huh7, PLC). **(D)** Immunofluorescence staining for FGF9 (green) and alpha-smooth muscle actin (alphaSMA; red) of primary human hepatic stellate cells. Nuclei were counterstained using DAPI (blue). **(E)** Correlation of FGF9 and alpha-sma mRNA expression in human HCC tissues (*n* = 14). **(F)** Representative image of immunofluorescence staining for FGF9 (green) and alphaSMA (red) in a human HCC tissue section. Nuclei were counterstained using DAPI (blue).

First, we systematically analyzed the expression of paracrine FGFs (subfamilies 1 (FGF1 and FGF2), 4 (FGF4, FGF5 and FGF6), 7 (FGF3, FGF7, FGF10 and FGF22), 8 (FGF8, FGF17, FGF18) and 9 (FGF9, FGF16, FGF20)) in activated human HSC from 3 different donors and 4 HCC cell lines (Hep3B, HepG2, PLC and Huh7) using qRT-PCR analysis. Notably, mRNA expression levels of FGF1, FGF2, FGF5, FGF7 and FGF9 were more than 100-fold higher in HSC compared with HCC cells (Fig. 1A). To get further insight into the role of these FGFs in HCC, we assessed the correlation between their tumorous expression levels and survival of HCC patients using the “SurvExpress” Biomarker validation for cancer gene expression database^11^. Computational stratification into “low-risk” and “high-risk” patient groups (based on prognostic index) revealed significant overexpression of FGF9 as well as reduced overall survival of high- compared to low-risk groups in the TCGA Liver Cancer dataset (n = 381) (Fig. 1B) as well as in the LIHC-TCGA HCC (n = 361) and Hoshida Golub Liver GSE10143 (n = 162) datasets (Suppl. Fig. 1A,B). In contrast, no correlation was found between tumorous FGF1-, FGF2-, FGF5-, and FGF7-expression levels and survival in HCC patients (data not shown).

The high expression of FGF9 in HSC as compared with HCC cells and the strong correlation of high FGF9 expression in human HCC tissues with the survival of HCC patients prompted us to focus our further analysis on FGF9. Western blot analysis confirmed FGF9 expression in HSC while no FGF9 expression was detectable in the HCC cell lines (Fig. 1C). Immunofluorescence analysis showed a distinct FGF9-signal in cell-culture activated primary human HSC that colocalized with alpha-sma, a characteristic marker of activated HSC and stromal myofibroblasts^3,4^ (Fig. 1D). Analysis of tumor tissue samples from HCC patients revealed a significant correlation of the expression levels of FGF9 and alpha-sma (Fig. 1E). For comparison, FGF9 and alpha-sma mRNA expression levels were more than 10-fold higher in activated HSC compared with HCC-tissue levels (Suppl. Fig. 1C,D). Furthermore, immunofluorescence analysis showed co-localization of alpha-sma and FGF9 in human HCC tissues (Fig. 1F). In summary, these results indicate activated HSC/myofibroblasts as cellular source of FGF9 in HCC and suggest that enhanced FGF9 expression in HCC promotes tumor progression.

### Effects of FGF9 on tumorigenicity of HCC cells

**Figure 2:**
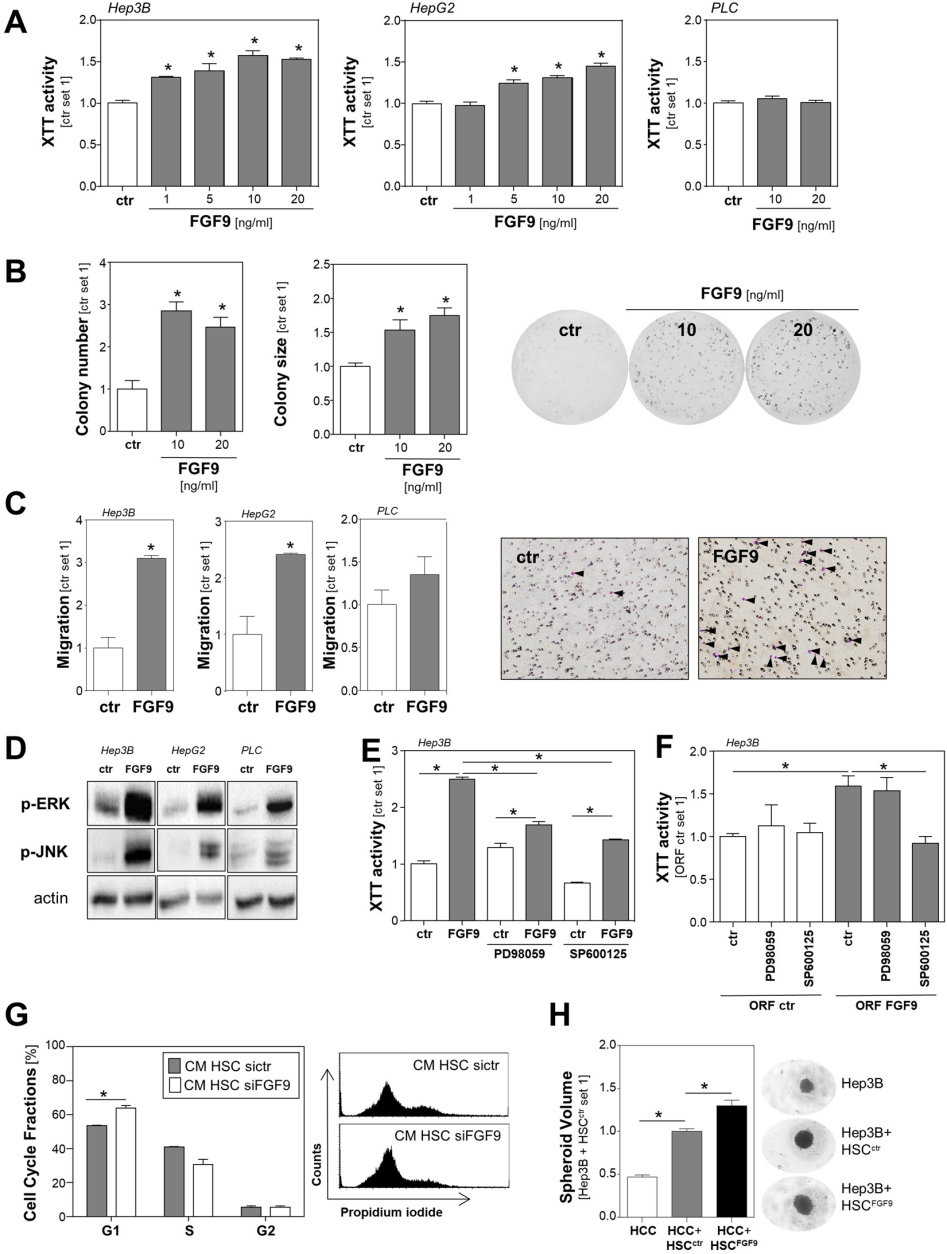
Effect of FGF9 on tumorigenicity of HCC cells. (**A**) Proliferation of HCC cell lines Hep3B, HepG2 and PLC stimulated without (ctr.) or with recombinant FGF9 (rFGF9) for 72 h. **(B)** Quantification of colony number and size (left and middle panel) and representative images (right panels) in anchorage-dependent clonogenic assays with Hep3B cells treated without (ctr.) or with rFGF9. **(C)** Migratory activity of HCC cells following 4 h treatment with rFGF9 (20 ng/ml; left panel) and representative images of Boyden chamber filters (right panel). Arrowheads indicate migrated cells. **(D)** Western Blot analysis of phosphorylated ERK and JNK1/2 in rFGF9 (20 ng/ml) treated and control cells. **(E)** Effects of PD98059 (a selective inhibitor of the MEK/ERK pathway; 10 µM) and SP600125 (JNK inhibitor; 10 µM) on rFGF9 (20 ng/ml)-induced proliferation of Hep3B cells. **(F)** Effects of PD98059 and SP600125 on proliferation of FGF9 overexpressing (HCC^FGF9^) and control (HCC^ctr^) Hep3B cells **(G)** FACS analysis of cell cycle fractions of HCC cells treated with conditioned media (CM) from HSC with siRNA-mediated FGF9 suppression (HSC-siFGF9) or HSC transfected with control siRNA (HSC-siCtr; left panel); representative images (right panel). **(H)** Volume of spheroids formed by Hep3B cells alone or mixed spheroids of Hep3B cells and control transfected HSC (HSC^ctr^) or Hep3B cells and HSC transfected with an FGF9 expression plasmid (HSC^FGF9^); representative microscopic images. (Analysis have been performed in triplicates; *p < 0.05).

Therefore, we wanted to analyze the effects of recombinant FGF9 (rFGF9) on tumorigenicity of human HCC cells in functional *in vitro* assays. With up to 20 ng/ml we applied similar FGF9 doses as used in previous *in vitro* studies^12–16^. Stimulation with rFGF9 caused a dose-dependent induction of the proliferation in Hep3B and HepG2 cells but not in PLC cells (Fig. 2A). Since FGF9 has been identified to be a high affinity ligand for mainly FGFR2 and FGFR3^17^, we compared the expression of these 2 FGF-receptors in HCC cell lines. While PLC cells showed comparable FGFR3 expression with HepG2 and Hep3B cells, FGFR2-expression was markedly reduced in PLC cells (Suppl. Fig. 1E,F). Thus, differences in FGFR2 expression might be a potential explanation for the varying FGF9 responsiveness regarding proliferation. In contrast, the very low FGF9 expression levels did not differ between the responsive (Hep3B and HepG2) and non-responsive (PLC) cells (Suppl. Fig. 1G) and further depletion of FGF9 with specific siRNA did not affect their proliferation (data not shown), further indicating that endogenous FGF9 expression in the different HCC cell lines has no significant impact on their proliferation.

Next, we assessed the impact of FGF9 on HCC cells in clonogenicity assays, which reflects also stem cell properties and cell survival of tumors cells. Here, stimulation with FGF9 induced the colony number as well as the colony size of Hep3B cells (Fig. 2B). In HepG2 cells, FGF9 stimulation did not affect the colony size and only the highest FGF9 dose induced the colony number (Suppl. Fig. 2A). In contrast, FGF9 dose dependently induced the colony size of PLC cells but not the colony number (Suppl. Fig. 2B). Analysis of the impact of FGF9 on the migratory activity of HCC cells in transwell Boyden chamber assays revealed that FGF9 significantly induced the directed migration of Hep3B and HepG2 cells but had only a slight effect on PLC cells (Fig. 2C). Together these data indicate, that generally FGF9 induces tumorigenic characteristics of HCC cells but there are qualitative and quantitative differences between different tumor cells. It appears, that differences in FGF-receptor expression may only partly explain this phenomenon, which has also already been described in other tumor entities. For example, Sun *et al*. described FGF9 did not induce proliferation but had an anti-apoptotic effect in gastric cancer cells^15^. Activation of mitogen-activated protein kinases (MAPK) is known to be associated with HCC proliferation, migration and stem cell properties including enhanced clonogenicity^1,18^. Therefore, we next analyzed the effect of FGF9 stimulation on the phosphorylation of extracellular-signal regulated kinase (ERK) and c-Jun N-terminal kinase (JNK) in HCC cells (Fig. 2D). FGF9 induced ERK- and JNK-phosphorylation in Hep3B, HepG2 as well as PLC cells (Fig. 2D). Incubation with PD98059, a specific MEK/ERK-pathway-inhibitor, significantly but not completely reduced the rFGF9-induced growth promoting effect in Hep3B cells. SP600125, a specific JNK-inhibitor, significantly reduced both basal as well as FGF9 induced proliferation (Fig. 2E). Still, relative to basal levels, FGF9 induced proliferation also in the presence of SP600125 in Hep3B cells (Fig. 2E). In HepG2 cells, PD98059 had no significant impact on the FGF9 induced growth promoting effect while SP600125 significantly reduced basal cell growth and also blunted rFGF9-induced proliferation (Suppl. Fig. 2C).

In addition to the effect of the stimulation with recombinant FGF9, we assessed the impact of overexpression of FGF9 in HCC cells. HCC cells were transfected with an FGF9-expression plasmid or empty vector as control (Suppl. Fig. 2D). FGF9-overexpression significantly increased the proliferation of HCC cells and this FGF9-induced proliferation was efficiently blocked by the JNK inhibitor SP600125 while ERK-inhibition showed no significant effect (Fig. 2F). Together, these data indicate that FGF9 inducing effect of the ERK- and JNK-pathways is at least in part responsible for the observed effects on different tumorigenic characteristics of HCC cells.

Next, we wanted to simulate the interaction between HSC (derived FGF9) and HCC cells *in vitro*. Once, HCC cells were incubated with either conditioned media (CM) from HSC with siRNA mediated FGF9 suppression or HSC transfected with control siRNA (Suppl. Fig. 2E). FACS analysis revealed that the growth promoting effect of CM from control HSC was significantly higher as compared with CM from FGF9-depleted HSC (Fig. 2G). Furthermore, we used a spheroid formation assay to assess the interaction between HCC and HSC. Here, we used a complementary approach and compared the effects of FGF9-overexpressing HSC (transfected with an FGF9-expression plasmid) and control HSC (transfected with empty vector) (Suppl. Fig. 2F). After 11 days, mixed HSC-HCC spheroids were significantly larger than spheroids formed by pure HCC cells (Fig. 2H). Still, spheroids of HCC-cells and FGF9 overexpressing HSC were significantly larger than spheroids of HCC-cells and control HSC (Fig. 2H and Suppl. Fig. 2G). Together, these findings indicate that FGF9 is not the only but a significant factor by which hepatic stellate cells promote the tumorigenicity of HCC cells.

### Effects of FGF9 on HCC cells in combination with sorafenib

**Figure 3:**
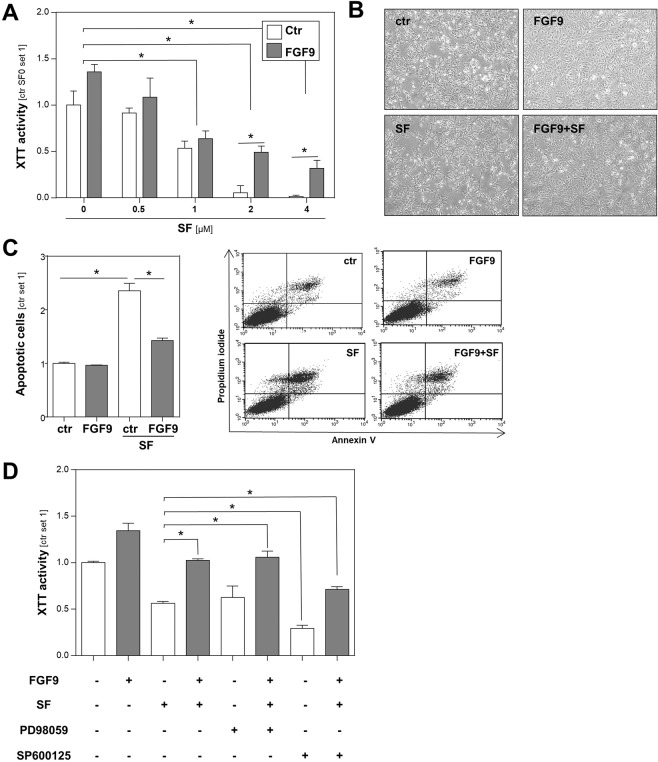
Effects of recombinant FGF9 on HCC cells in combination with sorafenib. (**A**) Proliferation of HCC cells treated with depicted doses of sorafenib (SF) and rFGF9 (20 ng/ml) for 48 h. **(B)** Corresponding microscopic images of HCC cells treated w/or w/o rFGF9 (20 ng/ml) and SF (2 µM). **(C)** Flow cytometric analysis of Annexin V-FITC and propidium iodide (PI) stained HCC cells following treatment with rFGF9 (20 ng/ml) and/or SF (2 µM). The right panel shows representative dot plots. **(D)** Effects of PD98059 (MEK/ERK inhibitor; 10 µM) or SP600125 (JNK inhibitor; 10 µM) on rFGF9 (20 ng/ml) induced sorafenib resistance of HCC cells. (Analyses have been performed in triplicates; *p < 0.05).

The multi-kinase inhibitor sorafenib also inhibits Raf/MEK/MAPK signaling and is currently the only clinically established pharmacological therapy for HCC. Therefore, we next wanted to analyze the effect of FGF9 on HCC cells in combination with the multi-kinase inhibitor sorafenib. Stimulation with rFGF9 significantly decreased the efficacy of sorafenib to inhibit proliferation and to induce cell death in HCC cells (Fig. 3A,B). PI/Annexin V flow cytometric analysis confirmed that rFGF9 inhibited sorafenib-induced apoptosis of HCC cells (Fig. 3C). Together, these data indicate that FGF9 enhances sorafenib resistance of HCC cells. Previous studies have linked JNK activation^19–22^ or ERK activation^23^ to sorafenib resistance. To determine whether FGF9 induced activation of ERK or JNK in HCC cells is responsible for the observed effect on sorafenib resistance, we repeated the analysis in the presence of the specific inhibitors PD98059 (MEK/ERK-pathway) or SP600125 (JNK-pathway). Interestingly, only SP600125 had a pronounced effect on rFGF9-induced sorafenib resistance (Fig. 3D). This indicates that JNK-activation is, at least in part, responsible for FGF9-induced sorafenib resistance of HCC cells and thus further suggests FGF9 and FGF9-induced signaling, respectively, as promising strategy in HCC.

### Modulation of FGF9 effects on HCC cells by FGFR-inhibitors

**Figure 4:**
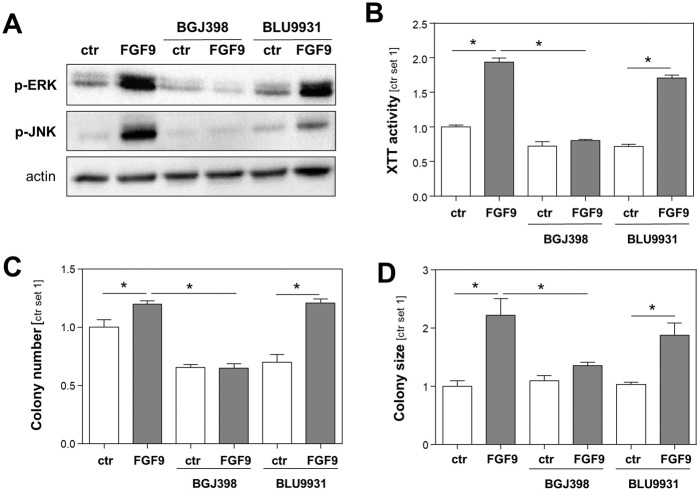
Effect of FGFR inhibitors on HCC cells. Effects of BGJ398 (a FGF receptor 1-3 inhibitor, 100 nM) and BLU9931 (a FGF receptor 4 inhibitor, 100 nM) on **(A)** rFGF9 (20 ng/ml)-induced phosphorylation of ERK and JNK1/2, **(B)** rFGF9 (20 ng/ml)-induced proliferation, and **(C)** colony number and **(D)** colony size anchorage-dependent clonogenic assays with Hep3B cells treated without (ctr.) or with rFGF9 (20 ng/ml). (Analysis have been performed in triplicates; *p < 0.05).

Therapeutic targeting of FGF-receptors (FGFR) with pharmacological inhibitors has shown promising results in preclinical HCC models^24^, and several FGFR inhibitors are in clinical trials to treat cancers harboring FGFR aberrations (e.g. ClinicalTrials.gov Identifier: NCT02965378). Therefore and to get further information on the receptors mediating the protumorigenic FGF9 effects in HCC cells, we compared the effects of the selective FGFR4 inhibitor BLU9931^25^ and the FGFR1/2/3 inhibitor BGJ398 (infigratinib)^25^ on HCC cells in the presence or absence of rFGF9. BGJ398 completely blocked the rFGF9-induced ERK- and JNK-phosphorylation in HCC cells (Fig. 4A). In contrast, BLU9931 had no effect on FGF9 induced ERK-phsophorylation and only slightly reduced JNK phosphorylation in HCC cells (Fig. 4A). Furthermore, BGJ398 completely inhibited the growth inducing effect of FGF9 in Hep3B cells while BLU9931 exhibited no significant effect (Fig. 4B). Moreover, the increased number and size of colonies formed by FGF9 stimulated HCC cells was blunted by BGJ398 while BLU9931 did not significantly affect the FGF9 effects on HCC cells in clonogenicity assays (Fig. 4C,D). Thus, while most preclinical and clinical studies (e.g. ClinicalTrials.gov Identifier: NCT02834780 and NCT03144661) focus on selective FGFR4 inhibitors^26^, our data indicate that rather FGFR1/2/3 inhibition is suitable to inhibit the protumorigenic FGF9 effects on HCC cells.

Future studies are needed to assess which of FGFR 1, 2 or 3 majorly contributes to the pro-tumorigenic effects of FGF9 on HCC cells and whether there may be individual differences, which could be used for targeted therapy with selective FGFR inhibition, which are in development^27^. Furthermore, FGF traps or anti-FGF antibodies^27^ could be exploited to specifically target FGF9 alone or in combination with other drugs, such as sorafenib. It is further tempting to speculate whether FGF9 expression levels could serve as biomarker for such therapeutic strategies. In any case, FGF9 expression levels appear as novel prognostic marker for survival of HCC patients. A recent study identified miR-140-5p as tumor suppressor in HCC and suggested that part of its inhibitory effect on tumor growth is mediated via suppressing FGF9, which these authors identified as miR-140-5p target in HCC cells^28^. Although our study indicates HSC/stromal myofibroblasts as major source of FGF9 in HCC, it may be that in a subset of HCCs also the tumor cells contribute to local FGF9 levels.

In this study, we focused on FGF9 effects on HCC cells and its impact on resistance to sorafenib. In addition, FGF/FGFR signaling has been implicated in angiogenesis and immune surveillance^25^. Therefore, it is possible that FGF9 exhibits further tumor promoting effects and leads to resistance to immune checkpoint or VEGF/VEGF receptor (VEGFR)-targeted agents, which needs to be addressed in future studies.

In summary, our study indicates that FGF9 derived from activated HSC enhances the tumorigenicity and therapy resistance of HCC cells embedded in a fibrotic microenvironment. These data may form the basis for future studies assessing the potential of FGF9 as prognostic biomarker and targeting of FGF9-FGFR signaling as a new treatment approach for patients with HCC.

## Materials and Methods

### Cells and cell culture

Primary human hepatic stellate cells (HSC) were isolated and cultured as described^29^. *In vitro* activation of HSC was achieved by cell culture on uncoated tissue culture dishes^29^. Tissue samples for cell isolation were obtained from patients undergoing partial hepatectomy for metastatic liver tumors. All experimental procedures were performed according to the guidelines of the non-profit state-controlled HTCR (Human Tissue and Cell Research) with informed patients’ consent^30^. Only those liver tissues judged as noncancerous by local pathologists were used for cell preparation. Further exclusion criteria were known liver disease or histologic evidence for liver fibrosis or inflammation in surrounding nontumorous liver tissue. Human HCC cell lines PLC (ATCC CRL-8024), Hep3B (ATCC HB-8064), HepG2 (ATCC HB-8065) and Huh7 (ATCC PTA-4583) were cultured as described^31^. Spheroid co-culture experiments of HCC cells and HSC were performed as described^31^.

For stimulation experiments, cells were treated with recombinant human FGF9 (R&D Systems, Minneapolis, MN, USA), sorafenib (Cayman Chemicals, Ann Arbor, MI, USA), PD98059 (inhibitor of the MEK/ERK pathway; Calbiochem, La Jolla, CA, USA), SP600125 (JNK inhibitor; Calbiochem), the FGF receptor 1-3 inhibitor BGJ398 (Selleckchem, Munich, Germany) or the FGF receptor 4 inhibitor BLU9931 (Cayman Chemicals).

SiRNA-induced knockdown of FGF9 was used applying an si-RNA-pool-FGF9 (siTOOLs Biotech GmbH, Planegg, Germany) and Lipofectamine RNAiMax transfection reagent (Life Technologies, Darmstadt, Germany) as described^32^. Overexpression of FGF9 protein was induced by transfection of a human FGF9 open reading frame (ORF) pcDNA3.1+/C-(K)DYK vector from GenScript (Piscataway, NJ, USA; CAT#: OHu27298) using LipofectAMINE plus method (Life Technologies, Darmstadt, Germany) as described^33^. An according empty control vector without the FGF9 ORF was used as control.

### Human tissue samples

Human HCC tissues and corresponding non-tumorous liver tissues were obtained from patients that underwent partial hepatectomy. All experimental procedures were performed according to the guidelines of the non-profit state-controlled HTCR (Human Tissue and Cell Research) with informed patients’ consent^30^. Sampling and handling of patient material were carried out according to the ethical principles of the Declaration of Helsinki.

### Immunofluorescence staining

Formalin-fixed primary human HSC and sections of formalin-fixed and paraffin-embedded HCC tissues were used for immunofluorescence staining applying anti-FGF9 antibodies (AF-273-NA, 1:25; R&D Systems, Minneapolis, MN, USA) and anti-alpha smooth muscle actin antibodies (ab32575, 1:500; Abcam, Cambridge, MA, USA) and standard protocols as described^34^. The following secondary antibodies were used: Alexa Fluor 488-conjugated donkey anti-goat IgG (A11055, 1:1,000; Invitrogen, Thermo Fisher Scientific, Waltham, MA, USA) and Cy3-conjugated donkey anti-rabbit IgG (711-165-152, 1:1,000; Jackson ImmunoResearch Laboratories, Inc., West Grove, PA, USA). Nuclei were counterstained using DAPI. For control of specificity, antibody diluent was applied instead of the primary antibody and rabbit and goat IgG (Sigma, Munich, Germany) were used as isotype controls. These control stainings showed no background signal (Suppl. Fig. 3).

### *In silico* analysis

The “SurvExpress-Biomarker validation for cancer gene expression” database (http://bioinformatica.mty.itesm.mx:8080/Biomatec/SurvivaX.jsp) was used for analysis of liver cancer datasets^11^.

### Analysis of mRNA expression

Total RNA was extracted using the PureLink RNA Mini kit (Ambion, Thermo Fisher Scientific, Waltham, MA, USA) following the manual’s instructions. Reverse transcription was performed using the Maxima First Strand cDNA Synthesis Kit (Thermo Scientific, Waltham, MA, USA). Quantitative real-time polymerase chain reaction was performed on a LightCycler 480 System (Roche Diagnostics, Mannheim, Germany) using specific sets of primers. Amplification of cDNA derived from β-actin or 18S rRNA was used for normalization of data.

### Protein analysis

Protein extraction and Western blotting were performed as described^31^ using the following primary antibodies: goat anti-FGF9 (AF-273-NA, 1:2,000; R&D Systems), rabbit anti-phospho-ERK (#9101, 1:1,000; Cell Signaling Technology, Danvers, MA, USA), rabbit anti-phospho-JNK (#9251, 1:1000; Cell Signaling Technology) and mouse anti-actin (MAB1501, 1:10,000; Merck Millipore, Billerica, MA, USA). Donkey anti-goat (sc-2020; 1:1,000; Santa Cruz Biotechnology, Heidelberg, Germany), chicken anti-rabbit (sc-2955; 1:10,000; Santa Cruz Biotechnology) and horse anti-mouse (Santa Cruz Biotechnology, sc-2005, 1:3,000) were used as secondary antibodies.

### Analysis of cell death, cell proliferation and cell cycle

For quantification of apoptosis, cells were simultaneously stained with FITC-conjugated Annexin V and propidium iodide using the Annexin V-FITC Detection Kit (PromoKine, PromoCell GmbH, Heidelberg, Germany) as described^35^. Proliferation of cells was determined using a colorimetric XTT assay (Roche Diagnostics, Mannheim, Germany) according to the manufacturer’s protocol^31^. Cell cycle fractions were analyzed by flow cytometry as described^36^.

### Clonogenic assay

Clonogenic assays were used to analyze anchorage-dependent colony formation and proliferation of cancer cells. The assay is based on the capability of single cells to grow into colonies and was described previously^32^.

### Analysis of cell migration

Migratory activity of HCC cells following treatment with FGF9 for 4 h was quantified using Boyden chamber assay as described^31^, with DMEM supplemented with 20% FCS attached to the bottom chamber.

### Statistical analysis

Statistical analysis was carried out using GraphPad Prism Software version 6.01 (GraphPad Software, San Diego, CA, USA). Data are shown as the mean ± standard error of the mean (SEM). Data sets were compared with analysis of unpaired Student’s t-test. A *p*-value <0.05 was considered statistically significant.

## Supplementary information

Supplementary Information.
