# Supplementary material for: Fibroblast Growth Factor 9 is expressed by activated hepatic stellate cells and promotes progression of hepatocellular carcinoma
Source: Sci Rep. 2020 Mar 11;10:4546. doi: 10.1038/s41598-020-61510-4 (PMC7066162; doi:10.1038/s41598-020-61510-4)

Supplementary Figures

Fibroblast Growth Factor 9 is expressed by activated hepatic stellate cells and promotes progression of hepatocellular carcinoma

Tatjana Seitz, Kim Freese, Peter Dietrich, Wolfgang Erwin Thasler, Anja Bosserhoff and Claus Hellerbrand

Suppl. Figure 1

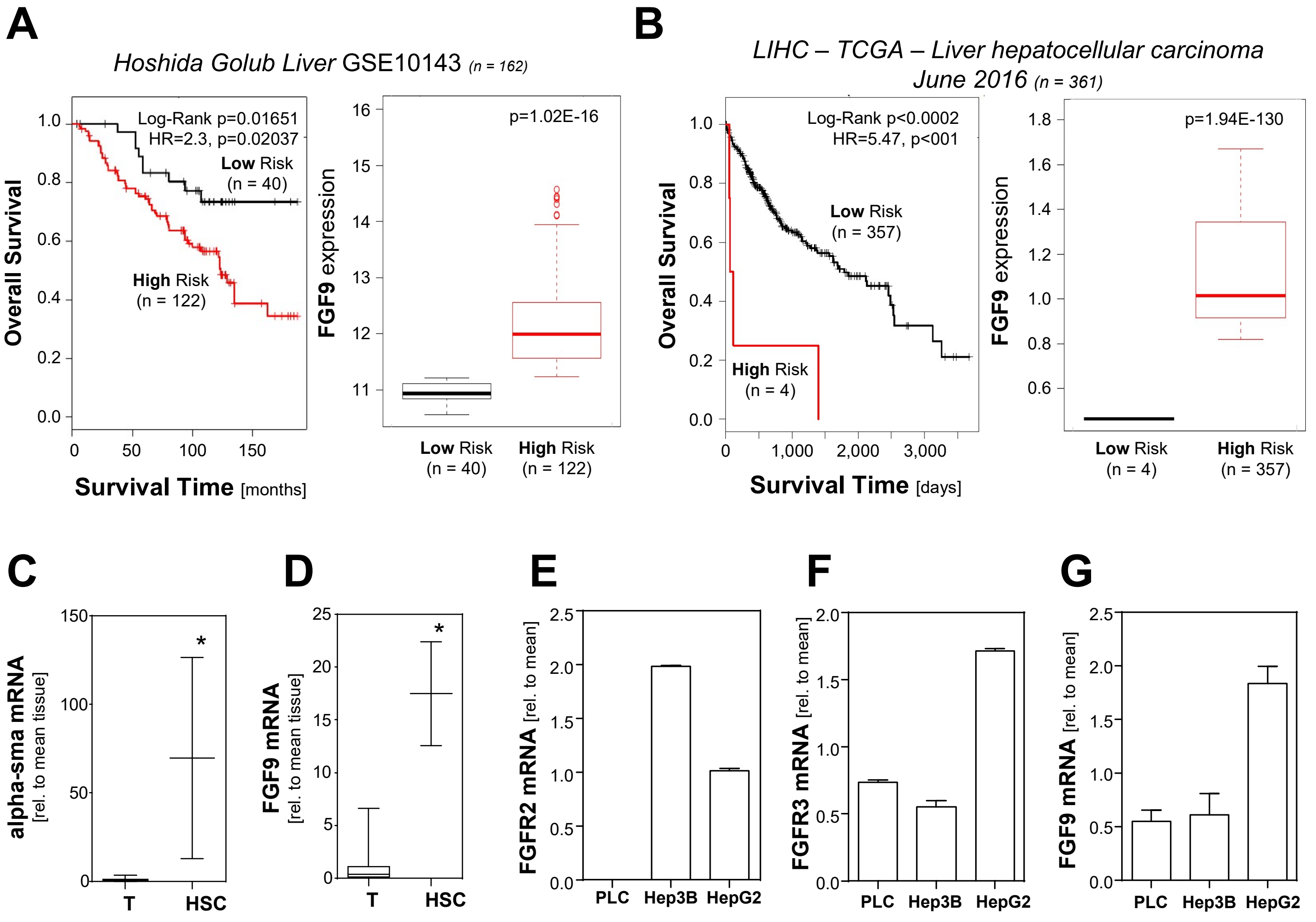

Suppl.Figure 1: "SurvExpress-Biomarker validation for cancer gene expression" database analysis of FGF9 expression (right panels) and corresponding Kaplan-Meier curve for overall survival (left panels) in **(A)** "Hoshida Golub Liver" and **(B)** "LIHC – TCGA – Liver hepatocellular carcinoma June 2016" datasets. Computational stratification of patients into "Low Risk" and "High Risk" groups was based on prognostic index and according to the "Maximized Risk Groups" algorithm. Analysis of **(C)** alpha-sma and **(D)** FGF9 mRNA expression in HCC tumor (T) tissue samples (n=18) compared to primary human activated hepatic stellate cells (HSC; from 2 different donors). Analysis of **(E)** FGFR2, **(F)** FGFR3 and **(G)** FGF9 mRNA expression in PLC, Hep3B and HepG2 cells. (*: p<0.05).

Supplementary Figures

Suppl. Figure 2

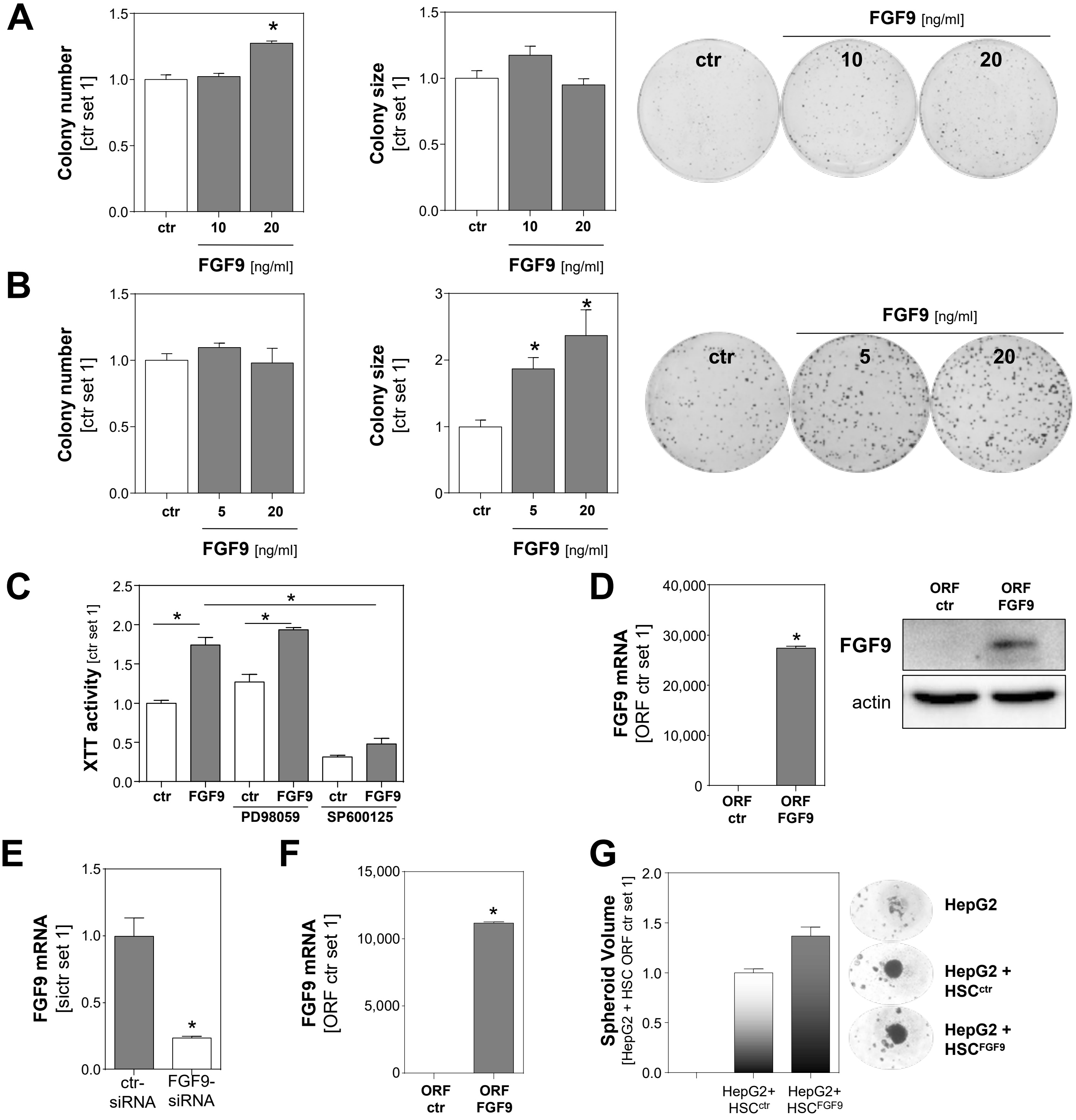

Suppl. Figure 2: Quantification of colony number and size (left and middle panel) and representative images (right panels) in anchorage-dependent clonogenic assays with **(A)** HepG2 cells and **(B)** PLC cells treated without (ctr.) or with rFGF9. **(C)** Effects of PD98059 (a selective inhibitor of the MEK/ERK pathway; 10 µM) and SP600125 (JNK inhibitor; 10 µM) on rFGF9 (20 ng/ml)-induced proliferation of HepG2. **(D)** FGF9 mRNA levels as quantified by qRT-PCR analysis (left panel) and FGF9 protein levels as quantified by Western blot analysis in Hep3B transfected with empty vector (ORF ctr) or a FGF9-expression plasmid (ORF FGF9). **(E)** FGF9 mRNA levels quantified by qRT-PCR in HSC transfected with control siRNA (ctr-siRNA) or si-RNA against FGF9 (FGF9-siRNA). **(F)** FGF9 mRNA levels quantified by qRT-PCR in HSC transfected with empty vector (ORF ctr) or an FGF9-expression plasmid (ORF FGF9). **(G)** Volume of mixed spheroids formed by HepG2 cells and control transfected HSC (HSC^{ctr}) or HepG2 cells and HSC transfected with an FGF9 expression plasmid (HSC^{FGF9}) after 11 days (left panel); representative microscopic images (right panel). Volume of mixed speroids of HepG2 cells and HSC^{ctr} was set as 1, as HepG2 cells alone did not form 3D structures. (All analysis have been performed at elast in triplicates; *: $p < 0.05$).

Supplementary Figures

Suppl. Figure 3

A B

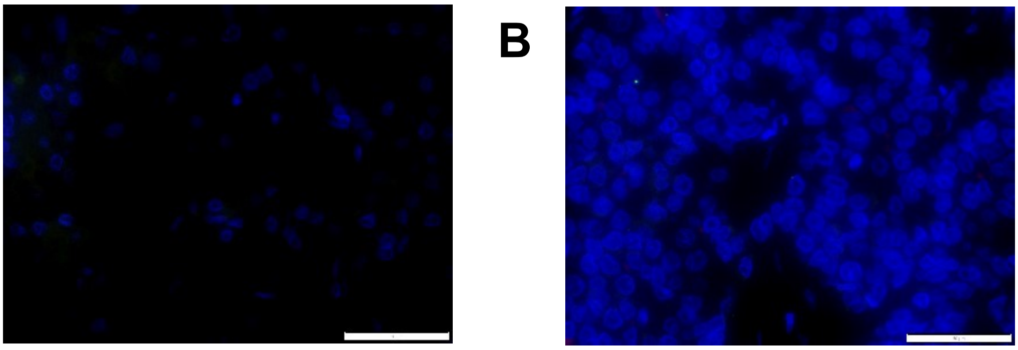

Suppl. Figure 3: Control images for alpha-sma and FGF9 immunofluorescence staining of HCC tissue. (A) No primary antibody and **(B)** isotype control. Nuclei were counterstained using DAPI.

Supplementary Files - *Original, uncropped blots*

Original, uncropped blots of Fig.1c

FGF9

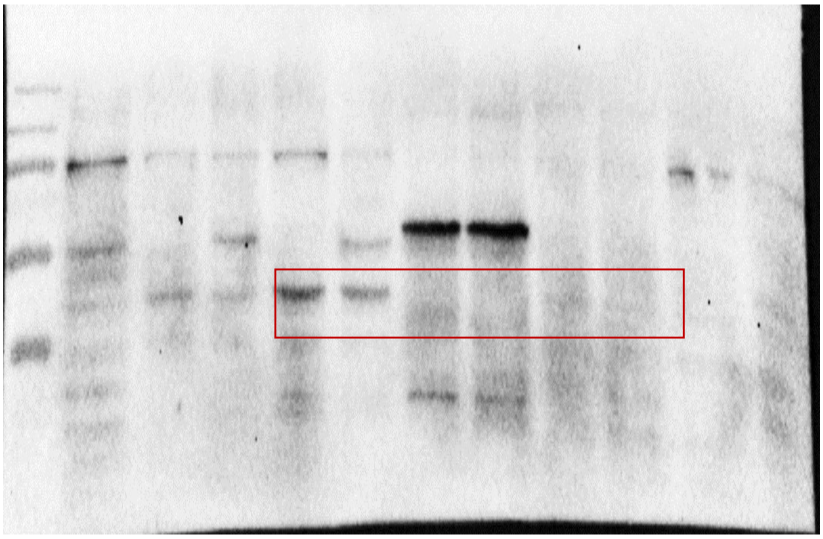

actin

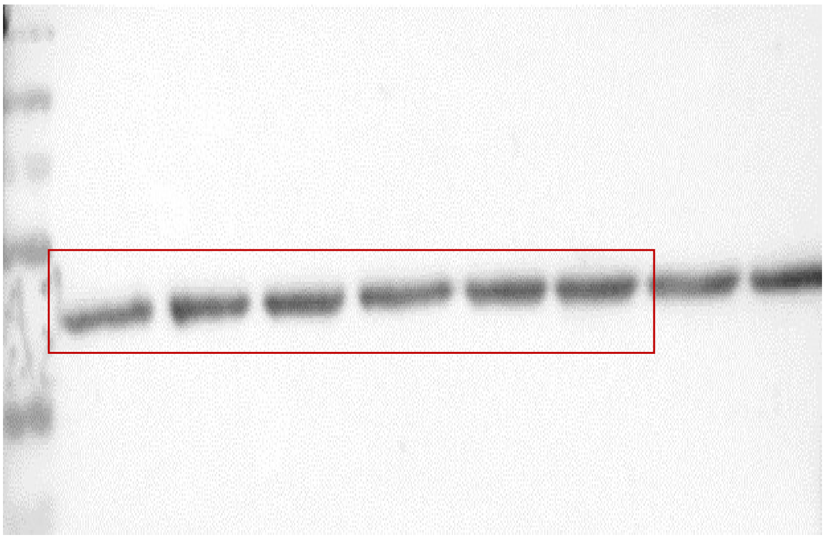

Supplementary Files - *Original, uncropped blots*

Original, uncropped blots of Fig.2d

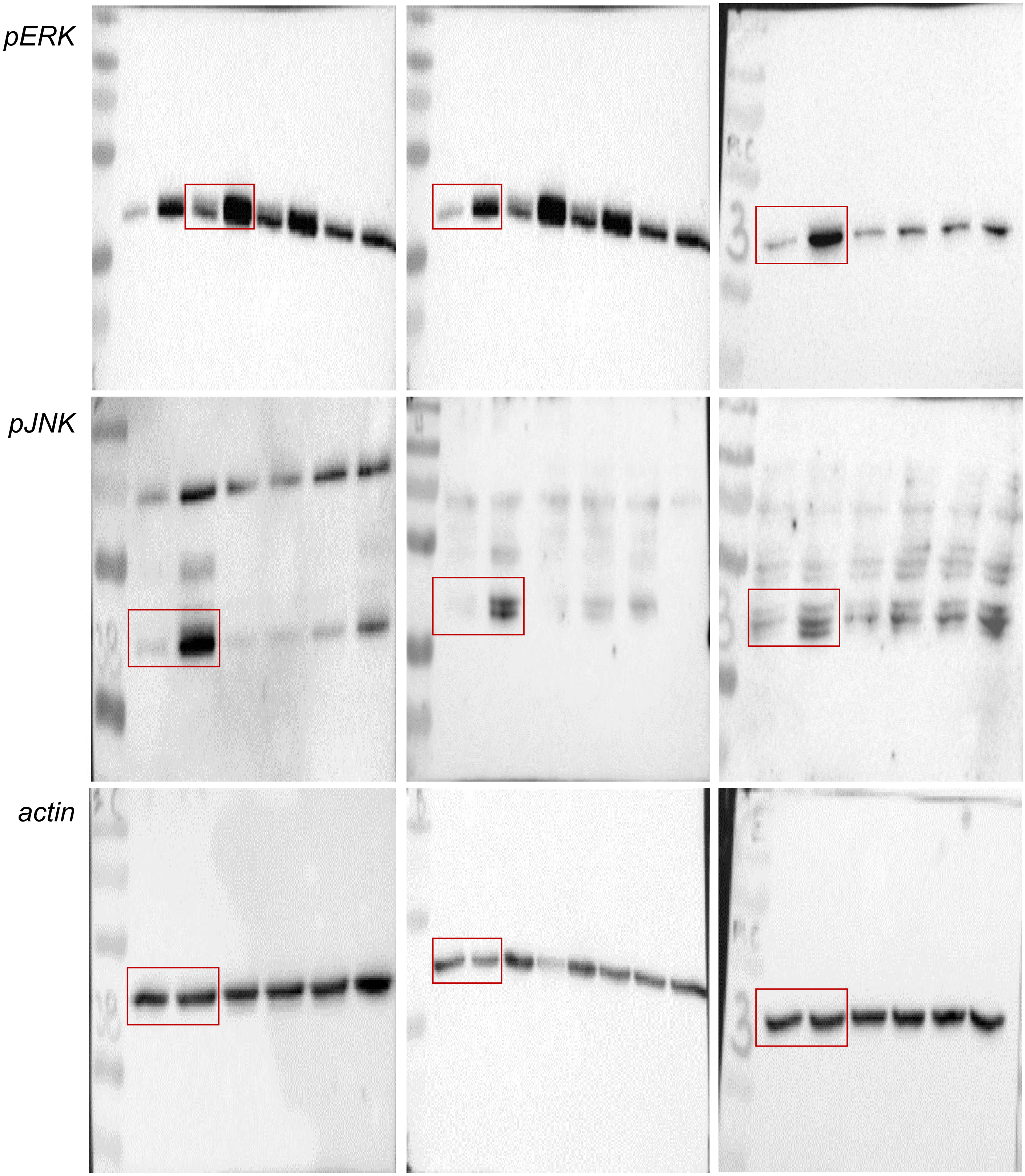

Supplementary Files - *Original, uncropped blots*

Original, uncropped blots of Fig.4a

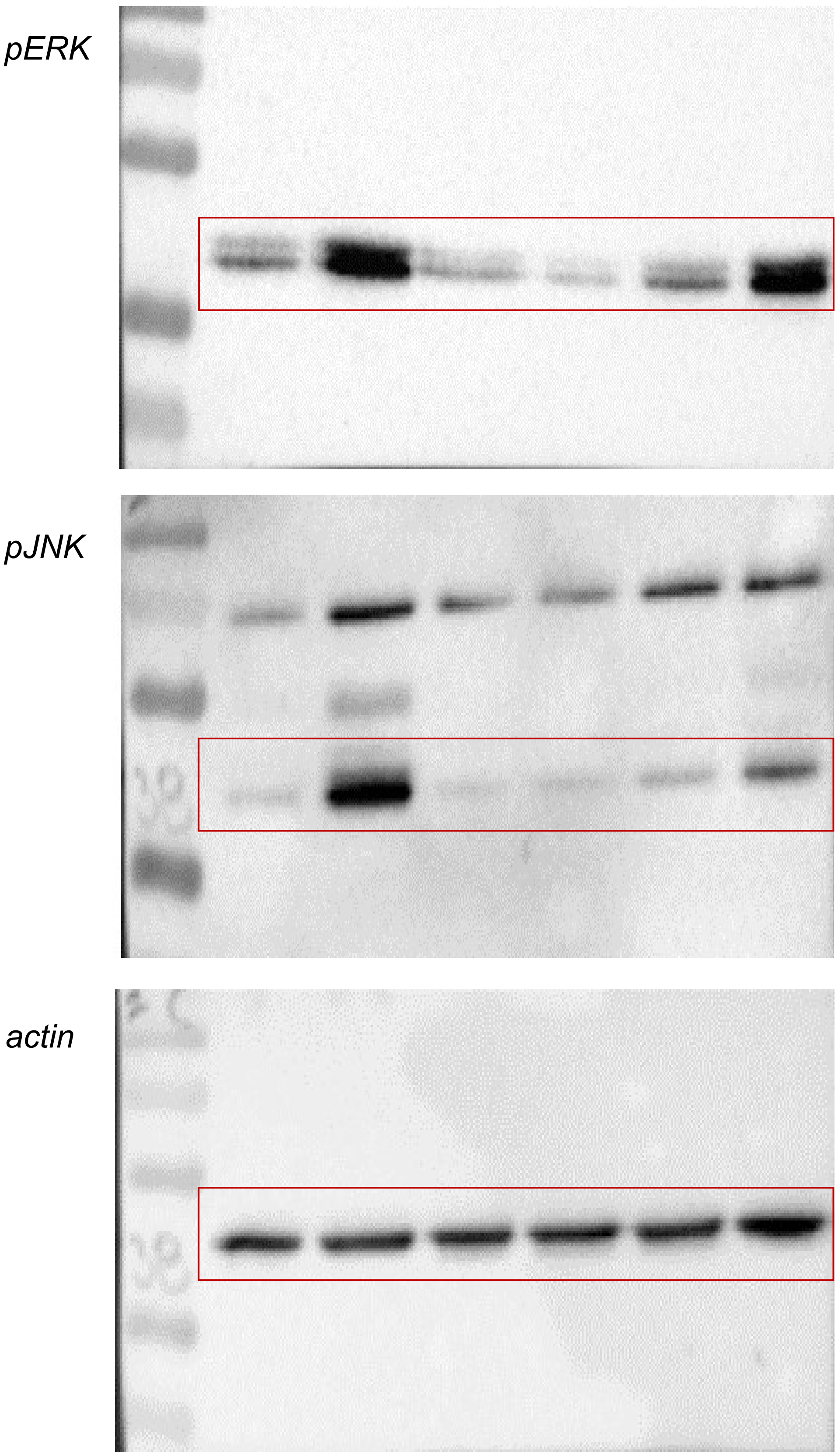

Supplementary Files - *Original, uncropped blots*

Original, uncropped blots of Suppl. Fig.2d

FGF9

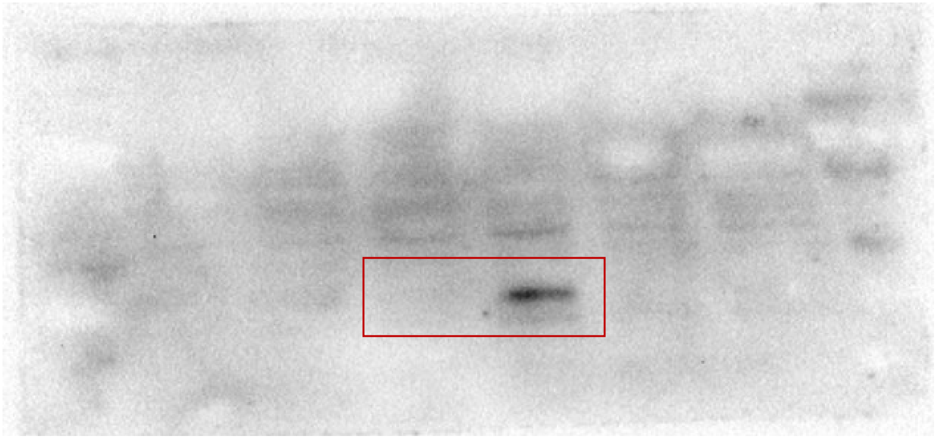

actin

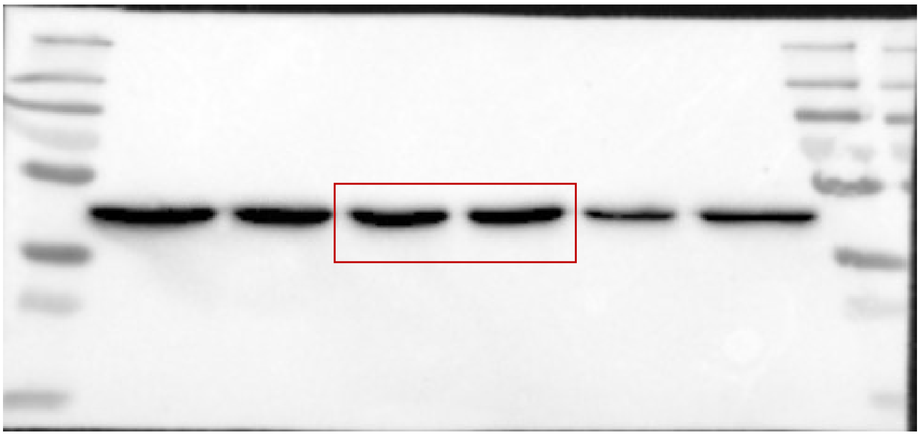

Supplement: Supplementary file 1 — Supplementary Information. [file 41598_2020_61510_MOESM1_ESM.pdf]
